# Comprehensive Enzymatic Conversion of Starch for the Food Industry

**DOI:** 10.3390/polym14214575

**Published:** 2022-10-28

**Authors:** Ekaterina Podgorbunskikh, Aleksandr Sapozhnikov, Timofei Kuskov, Daria Gurova, Anastasiia Kopylova, Aleksey Bychkov, Oleg Lomovsky

**Affiliations:** 1 Laboratory of Mechanochemistry, Institute of Solid State Chemistry and Mechanochemistry SB RAS, 18 Kutateladze Str., 630090 Novosibirsk, Russia; 2 Faculty of Business, Novosibirsk State Technical University, 20 Prospekt K. Marksa, 630073 Novosibirsk, Russia; 3 Department of Natural Sciences, Novosibirsk State University, 2 Pirogova Str., 630090 Novosibirsk, Russia

**Keywords:** tapioca starch, comprehensive enzymatic conversion, biodegradable packaging material, nano-sized starch particles, nano starch-based films, glucose syrup, recrystallization

## Abstract

This study demonstrated the feasibility of comprehensive enzymatic conversion of starch for non-waste applications in food industry. Enzymatic conversion of starch gives rise to nano-sized particles that can be used for manufacturing biodegradable and edible packaging materials and glucose syrup for replacing sugar in confectionery formulations. The 96 h enzymatic hydrolysis yielded starch nanoparticles smaller than 100 nm. Films based on nano-sized starch particles have promising physicochemical properties for manufacturing biodegradable and edible packaging materials. Such properties as reduced moisture content, increased homogeneity, crystallinity, and high initial thermal stability improve the mechanical and performance characteristics of the final food packaging materials. During film formation from starch subjected to preliminary mechanical amorphization, the polymer chain is recrystallized. The *C*-type crystal structure of starch is converted to the *B*-type structure. The supernatant obtained by starch hydrolysis can be used for producing glucose syrup. The resulting glucose syrup can be used as a sugar substitute in production of confectionery products. No objective technological differences in properties of glucose syrup obtained by comprehensive conversion of starch and the commercially available glucose syrup derived from sucrose were revealed.

## 1. Introduction

In the current society there is an ever-increasing unmet demand for biodegradable materials from renewable sources with the potential to be used for food storage and transportation. The most promising raw materials for obtaining such materials are natural polysaccharides, such as cellulose, starch, and chitosan [1,2,3]. Packaging materials based on biopolymers often need additional modification and improvement of performance characteristics. In addition, one should not forget about the ecological and economic part of the issue in order to create new technologies for food production, for example, waste-free comprehensive conversion of plant raw materials.

The important practical properties of starch, such as its availability and abundance, renewability, ability to be completely biologically degraded, low allergenicity, and low cost (much lower compared to cellulose) have been drawing researchers’ attention to the development of novel applications of starch in food and chemical industries, as well as bioengineering and materials science [4,5,6,7,8,9,10,11].

From the chemical standpoint, starch consists of two polysaccharides: linear amylose and branched amylopectin linked so as to form granules with an amorphocrystalline structure [12,13]. There exist three polymorphic modifications of starch (*A*-, *B*-, and *C*-types), which are found in different crops and slightly vary in terms of reactivity [12,13,14,15]. Along with carbohydrates, the commercially available starch samples also contain a small amount of lipids (up to 0.80 wt.%) and proteins (up to 0.40 wt.%), as well as 10–15% of moisture absorbed from the ambient air under normal conditions [16].

As an alternative to materials made from non-renewable resources, biodegradable (and even edible) polymeric materials (films, capsules, and coatings) based on polysaccharides are the subject of the rapidly developing trend in materials science [17,18]. The performance characteristics of materials produced from native starch have substantial drawbacks that limit their ubiquitous use and implementation (poor mechanical, thermal, and barrier properties, as well as high hygroscopicity) [19,20]. The quality of biodegradable packaging materials can be significantly improved by pre-modification of starch [21], cross-linking [22], or doping with nano-sized additives. However, doping with nano-sized particles (e.g., starch, cellulose, or chitin nanoparticles) as fillers can be regarded as the most promising approach that allows one to improve properties of polymeric packaging materials. The prevalence of surface properties over the bulk ones, which is typical of nano-sized objects, makes it possible to enhance mechanical properties (e.g., tensile strength) and thermal stability, as well as to control barrier properties (e.g., reduce vapor permeability) and biodegradation rate [23,24,25,26,27].

The process of nanoparticle production can be carried out according to two basic principles: “top-down” and “bottom-up” [28], which are carried out by the main groups of methods such as chemical (acid and/or enzymatic hydrolysis or their combination, renewal/micro perception) and physical methods (grinding, high-pressure homogenization, electrospray, gamma irradiation, and ultrasonic treatment) [28,29].

The basis of the “top-down” method of producing nanoparticles is the destruction of bulk material to obtain nanoparticles, and the “bottom-up” method is carried out through the growth-up and aggregation of molecules [28]. The most widely implemented method for preparation of starch nanoparticles (“top-down”) involves acid hydrolysis, which makes it possible to remove more reactive amorphous regions from the supramolecular structure [30]. Despite the greater research on acid hydrolysis, the use of enzymatic treatment is more promising, since it solves a number of problems associated with the use of acids, low yields of acid hydrolysis, which does not selectively disrupt not only amorphous regions of starch, but also crystalline ones [31,32]. Enzymatic hydrolysis makes it possible to realize both directions in the preparation starch nanoparticles. Enzyme treatment without gelatinization [31,33] refers to the “top-down” method of obtaining starch nanoparticles, while enzymatic hydrolysis followed by crystallization allows the “bottom-up” method to be implemented [28].

Along with fabrication of nanoparticles, enzymatic hydrolysis allows simple and inexpensive production of glucose syrup, which is in-demand not only as a confectionery ingredient in the food industry but also as feedstock for obtaining higher-value products in bioengineering [34,35,36,37,38].

This study focused on enzymatic hydrolysis enabling non-waste conversion of starch for the food industry that (1) yields nano-sized starch particles for producing biodegradable and edible packaging materials and (2) allows one to produce glucose syrup to be used as sugar substituent in confectionery formulations.

## 2. Materials and Methods

### 2.1. Materials

The reagents used in this study were as follows: tapioca starch (State Standard GOST 32159-2013; ZAO Garnec, Vladimir, Russia), distilled and bidistilled water, potassium ferricyanide (III) (SIGMA-Aldrich, Moscow, Russia), D(+)-glucose (99%, SIGMA-Aldrich, St. Quentin Fallavier, France), and glycerol (≥99.5%, SIGMA-Aldrich, Moscow, Russia). The enzymatic cocktail “Amylosubtilin G3x” (Technical specifications TU 9291-015-13684916-07, Sibbiopharm Ltd., Berdsk, Russia) was used; its biocatalytic activity on model substrates was as follows: α-amylase, not less than 1500 units/g; β-glucanase, up to 500 units/g; and xylanase, up to 100 units/g.

### 2.2. Particle Size of Native Starch

The particle sizes of native starch were measured on a CAMSIZER X2 optical analyzer (Retsch GmbH, Haan, Germany) with a detection threshold of 0.8–8000 μm and compressed air dispersion module (pressure 50 kPa). The average particle size was calculated using the image analysis method in compliance with ISO standard 13322-2:2006.

### 2.3. Mechanical Treatment

Tapioca starch was subjected to mechanical treatment in an AGO-2 water-cooled planetary ball mill (ISSCM SB RAS, Novosibirsk, Russia) (grinding body acceleration, 200 m/s^2^; nominal motor power, 1.1 kW). Steel balls (diameter, 5 mm; weight, 200 g) were used as grinding media. The weight of treated material was 10 g; the duration of mechanical treatment was 600 s.

### 2.4. Producing Starch Nanoparticles and Glucose Syrup

Enzymatic hydrolysis of starch (moisture content in the native starch, 10.5%) was carried out at a temperature of 55 °C and stirring speed of 120 rpm. To perform hydrolysis, starch was suspended in an enzyme solution (10 g of starch + 250 mL of the enzyme cocktail solution). To collect glucose syrup and starch nanoparticle samples, the reaction was stopped by rapid cooling to 4 °C after 6, 12, 24, and 96 h. The supernatant was then separated by centrifuging (12,000 rpm/10 min); the precipitate was washed several times with distilled water until neutral pH, frozen, dried by freeze dehydration, and weighed to determine the yield.

The reducing sugar content was measured using the Hagedorn–Jensen ferrocyanide method [39]. D(+)-Glucose and potassium hexacyanoferrate (III) were used to plot a calibration curve at λ = 420 nm with respect to water. The resulting supernatant was partially boiled off to obtain glucose syrup.

### 2.5. The Zeta Potential and Particle Size of Starch Nanoparticles

The zeta potential and the average particle size of the native and nano starch were determined using a Photocor Compact-Z (Photocor Ltd., Moscow, Russia) at a wavelength of 635.5 nm and 450 nm, respectively. For this purpose, 0.01% suspension of starch nanoparticles in bidistilled water was prepared; the resulting solution was dispersed at 25,000 rpm for 20 min on a T18 digital ULTRA TURRAX ultradisperser (IKA, Staufen, Germany).

### 2.6. Film Preparation

Suspensions (3 wt. %) of native starch were subjected to gelatinization at 80 °C for 30 min. Next, nano-sized starch particles (5%) were added to the gellified solutions of native starch. The resulting solution was kept at room temperature under stirring at 300 rpm for 30 min, and vacuum degassing was performed. The degassed solutions were poured into round molds 35 mm in diameter (0.36 g/cm^2^) and dried in a drying oven at 35 °C for 24 h to obtain films.

### 2.7. Moisture Content

The moisture content of native and nano starch-based composite films was determined on a WPS 50 SX moisture analyzer (Radwag, Radom, Poland) using a halogen lamp as a heating element. Drying to constant mass (mass loss during 3 min drying being less than 2 mg) was performed.

### 2.8. Film Thickness

Film thickness was measured using an MK 0–25 mm micrometer (Calibre, Moscow, Russia) (State Standard GOST 6507-90). Film thickness at 10 different spots was measured for each sample.

### 2.9. X-ray Diffraction Analysis

X-ray diffraction analysis was carried out on a D8 Advance X-ray powder diffractometer (Bruker, Karlsruhe, Germany) with CuKα radiation at a wavelength of 1.5406 Å in the Bragg–Brentano reflection geometry, at a scan step of 0.0195° in the 2θ angle range = 3°–60°.

The crystallinity index (*CrI*) was calculated as the ratio between the area of the crystalline phase and the total area under the XRD curve using Equation (1) [40]:

(1) $$CrI=\frac{S_{\mathrm{cr}.\mathrm{phase}}}{S_{\mathrm{total}}}*100\%,$$

where $S_{\mathrm{cr}.\mathrm{phase}}$ is the area of the crystalline phase and $S_{\mathrm{total}}$ is the total area below the XRD pattern curve.

### 2.10. Scanning Electron Microscopy (SEM)

The particle morphologies of native tapioca starch, native starch film, and starch film + 5% starch nanoparticles were studied by scanning electron microscopy on a TM-1000 microscope (Hitachi, Tokyo, Japan) at an accelerating voltage of 15 kV. A gold coating was deposited onto the sample surface to remove the accumulated charge using a JFC-1600 Auto Fine Coater (Jeol, Tokyo, Japan) (ion current, 30 mA; spray time, 40 s).

### 2.11. Differential Scanning Calorimetry (DSC)

Differential scanning calorimetry analyses were carried out in argon gas atmosphere on a DSC 204 F1 (Netzsch, Weimar, Germany). Approximately 2 mg of native tapioca starch and starch-based films was weighed in hermetic pans in order to avoid water loss. An empty hermetic pan was used as a reference. Samples were heated from 25 to 250 °C at a heating rate of 10 °C/min in the argon atmosphere.

### 2.12. Preparing Confectionery Products Using the Resulting Glucose Syrup

The following ubiquitously available ingredients were used to prepare confectionery products (gingerbread): wheat flour (top-grade, State Standard GOST 26574-2017; Aleyskzernoprodukt Ltd., Aleysk, Russia), flaxseed flour (Technical specifications TU 9146-004-31496822-2009; Khlebzernoprodukt Ltd., Taganrog, Russia), buckwheat flour (State Standard GOST 31645-2012; Kompas Zdorovya Ltd., Novosibirsk, Russia), corn flour (State Standard GOST 14176-69; Petersburg Mill Plant Ltd., Saint-Petersburg, Russia), glucose syrup (State Standard GOST 33917-2016; Ruskvas Ltd., Lytkarino, Russia), experimental glucose syrup, chicken eggs (State Standard GOST 31654-2012; JSC “Poultry Farm Evsinskaya”, Novosibirsk region, Evsino station, Russia), butter 72.5% fat (State Standard GOST 32261-2013; “Prostokvashino trademark”; JSC “Danone Russia”, Moscow, Russia), “Motley grass” honey (State Standard GOST 19792-2017; LLC “Tradehouse Voyal”; Novosibirsk, Russia), baking soda (State Standard GOST 32802-2014; JSC “Bashkir Soda Company”, Sterlitamak, Russia), dough baking powder (State Standard GOST 32802-2014; “Trapeza” trademark, LLC “Novosibirsk Food Factory”, Novosibirsk, Russia).

To bake gingerbread according to the basic commonly used formulation [41], the prepared sugar, water, honey, and eggs were stirred during 6–10 min. Baking soda, baking powder, softened butter, and top-grade flour were then added, and the dough was kneaded for 4–12 min. The dough was rolled out to a thickness of 8–10 mm, and the gingerbread was shaped and baked at 200–240 °C for 10–15 min. In the analyzed gingerbread samples, the commercially available glucose syrup was replaced at the first stage with glucose syrup produced by starch hydrolysis, without any other changes to the formulation made.

### 2.13. Quality Evaluation of the Confectionery Products

Gingerbread samples were evaluated according to their appearance, taste, smell, color, and consistency in compliance with the State Standard GOST 31986-2012. Each of the sensory evaluation indicators (appearance, taste, smell, color, and consistency) was evaluated on a 5-point scale, where 5 points is the highest score and 1 point is the lowest score.

### 2.14. Statistical Analysis

In each assay, repeats were performed at least 3 times on different representative samples. Data were expressed as mean ± standard deviation (SD).

## 3. Results and Discussion

### 3.1. Physicochemical Properties of Native Starch

**Figure 1:**
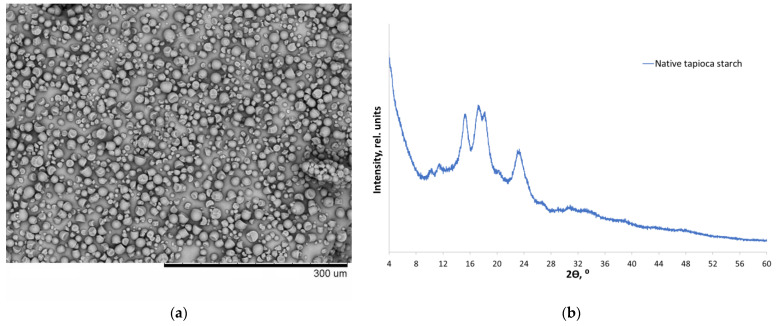
The SEM morphology of the granules of native tapioca starch (**a**) and the X-ray powder diffraction pattern of native tapioca starch (**b**).

Tapioca starch had a moisture content of 7.5% and an average particle size of 17 ± 1 μm. Granules of native tapioca starch had a predominantly spherical morphology, with numerous polyhedral fragments (Figure 1a). Tapioca starch granules had a mixed *C*-type crystal structure (Figure 1b). Thermal properties of native starch were identified: the onset temperature (T_1_) 293.6 ± 0.2; the peak temperature (T_2_) 298.1 ± 0.3; the endset temperature (T_3_) 314.0 ± 0.5; and the enthalpy of phase transition (ΔH) −20.61 ± 0.8 J/g.

### 3.2. Production of Nano-Sized Starch Particles and Glucose Syrup

The enzymatic hydrolysis reaction was fully stopped after 6, 12, 24, and 96 h for collecting the supernatant to produce glucose syrup and characterize starch particles after the hydrolysis (Table 1). Table 1 shows that the yield of reducing sugars suitable for producing confectionery glucose syrup remained almost unchanged after 24 h. The increased crystallinity index of starch after the enzymatic hydrolysis also indicates that the regions of polymeric chain of starch were hydrolyzed. Similar results were obtained for corn starch [25].

However, the depth of enzymatic hydrolysis sufficient for the formation of starch nanoparticles was reached after 24 h. The yield of starch nanoparticles after 96 h hydrolysis was 34 ± 2%. The zeta potential was +5.6 mV; its low value indicates that interparticle repulsion was low.

### 3.3. Preparing Native and Nano Starch-Based Films from Tapioca Starch

**Figure 2:**
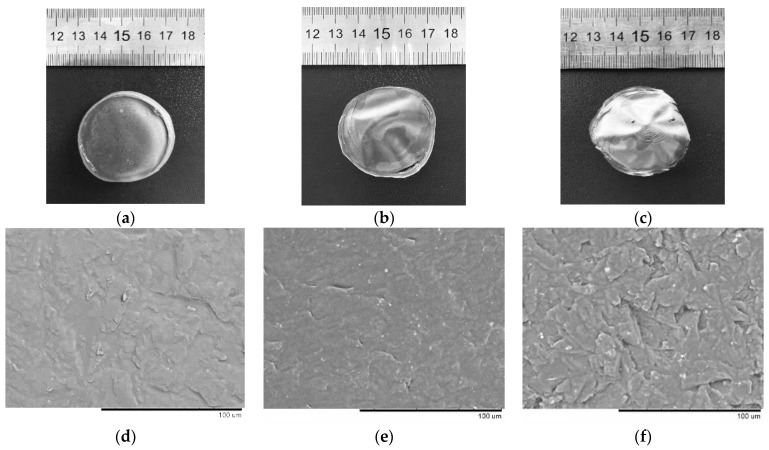
The photo (**a**) and SEM image (**d**) showing the native starch film; the photo (**b**) and SEM image (**e**) of the amorphous starch film; and the photo (**c**) and SEM image (**f**) of nano starch-based film.

Figure 2 shows the morphology of films based on native starch, amorphized starch, and composite films doped with 5% nano-sized starch particles. The films prepared from native starch were semitransparent and textured; after doping with nanoparticles, the films became less transparent and had a smoother surface (Figure 2a,c). The films prepared from amorphized starch had the smoothest and most homogeneous morphology; film transparency significantly increased (Figure 2b).

The SEM images of the films prepared from native starch show that they have a textured surface (Figure 2d). The amorphous starch films had a homogeneous and regular microstructure without inclusions (Figure 2e). The surface of the films doped with 5% nanoparticles became rougher, but there was neither obvious nanoparticle aggregation nor phase separation.

### 3.4. Physicochemical Properties of Films Prepared from Native Tapioca Starch and Nano Starch

Table 2 summarizes the film thickness, moisture content, and the crystallinity index. The onset temperature (T_1_), the melting peak maximum (T_2_), the endset temperature (T_3_), and the enthalpy (ΔH) of thermal phase transitions were also determined to characterize thermal properties.

The thickness of the films prepared from pre-amorphized starch changed twofold, and the thickness of the films doped with nano-sized starch particles decreased threefold; film homogeneity increased. Moisture content in the films prepared from amorphous starch and in composite films based on nanoparticles differed 1.5- and 2-fold, respectively.

**Figure 3:**
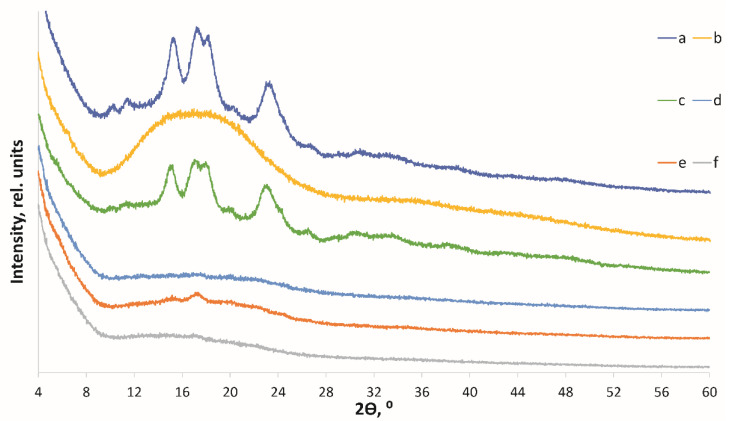
The XRD pattern of native tapioca starch (**a**); amorphous starch (**b**); nano-sized starch (**c**); native starch-based film (**d**); amorphous starch-based film (**e**); and nano starch-based film (**f**).

As films were prepared from amorphized starch (Figure 3b), the polymeric chain was recrystallized during heterogeneous treatment (under the exposure to an aqueous solution and elevated temperatures). Meanwhile, the crystal structure of the native *C*-type starch was converted to the *B*-type (Figure 3d). The observed phenomenon is typical of amorphocrystalline polymers such as cellulose [25,42].

Doping with starch nanoparticles increased the crystallinity of the resulting films from 11% to 18% (Figure 3b). The reduced sensitivity of nanoparticle-based films to swelling (moisture content), as well as the increased film homogeneity and crystallinity enhance the mechanical and performance characteristics of the final food-packaging materials.

The thermal properties of tapioca starch-, amorphous starch-, and nano starch-based films are summarized in Table 2. Slight differences in the peak onset temperatures (T_1_), the peak temperatures (T_2_), and the peak endset temperatures (T_3_) were observed between the all films. The enthalpy for amorphous tapioca starch-based film was −53.6 J/g, which may indicate greater homogeneity (fewer structural defects) and greater thermal stability. The high temperatures of endothermal peak for tapioca starch, native starch films, amorphous starch film, and nanoparticle-containing films can be attributed to the fact that starch gelatinization during the film production was completed and that tapioca starch has a higher crystallinity (41%); they also demonstrate that granules have a higher structural stability, thus resulting in higher thermal stability [24,43].

### 3.5. Producing Glucose Syrup for Confectionery Products from the Supernatant

To produce glucose syrup, the supernatant collected after enzymatic conversion of starch was concentrated in a water bath until reaching a thick consistency, which is typical of commercially available molasses. A viscous fluid of rich caramel color, with sweet odor and taste, was obtained.

**Figure 4:**
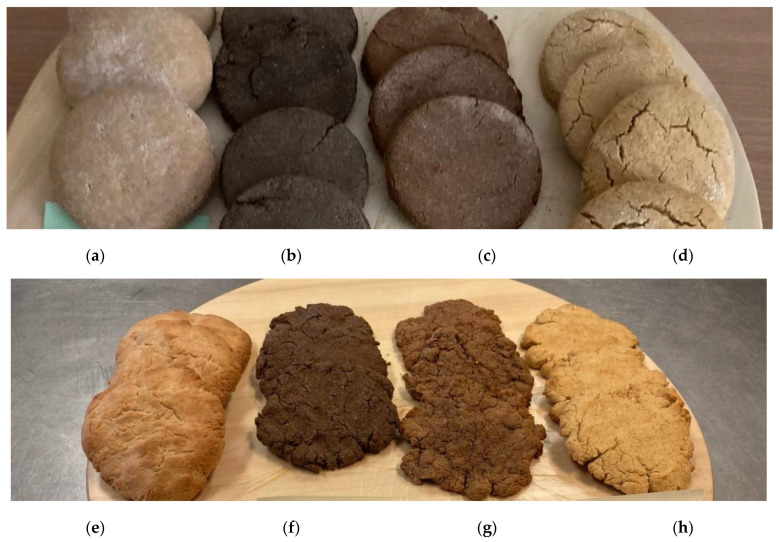
The gingerbread samples prepared using commercial glucose syrup and wheat (**a**), flaxseed (**b**), buckwheat (**c**), and corn (**d**) flour. The gingerbread samples prepared using the experimental glucose syrup and wheat (**e**), flaxseed (**f**), buckwheat (**g**), and corn (**h**) flour.

Gingerbread samples were baked to compare properties of the produced glucose syrup and the commercially available analog. The following gingerbread samples were prepared from wheat, flaxseed, buckwheat, and corn flour (Figure 4). The molasses obtained during sucrose production is conventionally used in the food industry. However, the long-term consumption of these molasses containing equal amounts of glucose and fructose can have an unfavorable effect on human health [44]. Therefore, glucose syrup produced from starch by hydrolysis is of interest [45]. The findings have been published demonstrating that consumption of glucose syrup instead of sucrose increases the body’s tolerance to glucose, thus normalizing the carbohydrate metabolism [46]. There are data showing that it is reasonable to use glucose syrup as a sugar substitute when producing various products [47,48].

**Figure 5:**
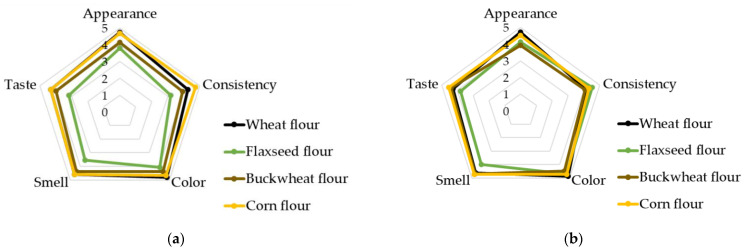
Sensory evaluation of the gingerbread samples for the samples prepared using the commercial glucose syrup (**a**) and the experimental glucose syrup (**b**).

The samples mainly differed in appearance, which can be explained by the insufficiently viscous consistency of the glucose syrup produced by enzymatic conversion of starch. These differences are not objective quality criteria and can be easily corrected using routine procedures for optimizing the composition of the components. The more important parameters, the results of comprehensive sensory evaluation (Figure 5), demonstrated that the samples were similar.

The sensory evaluation scale scores for all the samples were almost identical. The gingerbread made from corn flour had the greatest score: 23 points for the commercial sample of glucose syrup and 22.9 points for the experimental sample of glucose syrup. The gingerbread samples made from flaxseed flour had the lowest score: 17.8 points for the commercial sample of glucose syrup and 21.1 points for the experimental sample of glucose syrup, respectively. It indicates that the role of the source of glucose syrup as the end product is negligible and much less significant than the role of the main components (flour).

## 4. Conclusions

It was demonstrated for tapioca starch that starch nanoparticles and glucose syrup can be produced by enzymatic hydrolysis under mild conditions (without using buffer solutions). Long-term enzymatic hydrolysis (up to 96 h) gave rise to starch nanoparticles smaller than 100 nm at a high yield (~34%). The supernatant obtained after the enzymatic hydrolysis could be used to produce glucose syrup for substituting sugar in confectionery formulations (e.g., gingerbread) after only 24 h.

As amorphized starch films were formed, the polymeric chain was recrystallized due to swelling and exposure to high temperatures. The *C*-type crystal structure of the native starch became *B*-type.

Starch films made from native starch and the nano starch-based composite film (concentration, 5%) have promising properties for producing biodegradable and edible packaging materials. Such properties as the reduced moisture content, homogeneity, high crystallinity, and high initial thermal stability improve the mechanical and performance characteristics of the final food-packaging materials.

The supernatant can be used to produce sugar substitute (glucose syrup). The differences in properties of glucose syrup obtained by comprehensive conversion of tapioca starch and the commercially available glucose syrup can be mitigated using routine procedures for optimizing the confectionery formulations.

## Figures and Tables

**Table 1:** The yield of reducing sugars, crystallinity index, and particle size of tapioca starch after enzymatic hydrolysis.

Time, h	Yield Reducing Sugar, %	CrI, %	Average Particle Size, nm
0	-	41 ± 1	>1000
6	18 ± 1	52 ± 3	642 ± 40
12	40 ± 1	50 ± 2	428 ± 30
24	59 ± 2	49 ± 2	189 ± 17
96	64 ± 3	47 ± 2	66 ± 6

**Table 2:** Thickness, moisture, the crystallinity index, and thermal properties of native and nano starch-based films.

Sample	Film Thickness, µm	Moisture Content, %	CrI, %	T_1_, °C	T_2_, °C	T_3_, °C	ΔH, J/g
Native starch film	13.6 ± 1.4	31.8 ± 0.1	11.4 ± 0.4	290.3	296.3	300.8	−18.54
Amorphous starch film	7.0 ± 0.7	20.3 ± 0.1	18.5 ± 0.9	289.4	294.7	304.7	−53.60
Nano starch-based films	4.7 ± 0.7	14.9 ± 0.1	21.6 ± 0.7	289.8	292.7	297.6	−19.70

## Data Availability

Not applicable.
